# Transgene-Induced Gene Silencing Is Not Affected by a Change in Ploidy Level

**DOI:** 10.1371/journal.pone.0003061

**Published:** 2008-08-26

**Authors:** Daniela Pignatta, Brian Dilkes, Tadeusz Wroblewski, Richard W. Michelmore, Luca Comai

**Affiliations:** 1 Department of Plant Biology and Genome Center, University of California Davis, Davis, California, United States of America; 2 Department of Plant Sciences and Genome Center, University of California Davis, Davis, California, United States of America; Temasek Life Sciences Laboratory, Singapore

## Abstract

**Background:**

Whole genome duplication, which results in polyploidy, is a common feature of plant populations and a recurring event in the evolution of flowering plants. Polyploidy can result in changes to gene expression and epigenetic instability. Several epigenetic phenomena, occurring at the transcriptional or post-transcriptional level, have been documented in allopolyploids (polyploids derived from species hybrids) of *Arabidopsis thaliana*, yet findings in autopolyploids (polyploids derived from the duplication of the genome of a single species) are limited. Here, we tested the hypothesis that an increase in ploidy enhances transgene-induced post-transcriptional gene silencing using autopolyploids of *A. thaliana*.

**Methodology/Principal Findings:**

Diploid and tetraploid individuals of four independent homozygous transgenic lines of *A. thaliana* transformed with *chalcone synthase* (CHS) inverted repeat (hairpin) constructs were generated. For each line diploids and tetraploids were compared for efficiency in post-transcriptional silencing of the endogenous CHS gene. The four lines differed substantially in their silencing efficiency. Yet, diploid and tetraploid plants derived from these plants and containing therefore identical transgene insertions showed no difference in the efficiency silencing CHS as assayed by visual scoring, anthocyanin assays and quantification of CHS mRNA.

**Conclusions/Significance:**

Our results in *A. thaliana* indicated that there is no effect of ploidy level on transgene-induced post-transcriptional gene silencing. Our findings that post-transcriptional mechanisms were equally effective in diploids and tetraploids supports the use of transgene-driven post-transcriptional gene silencing as a useful mechanism to modify gene expression in polyploid species.

## Introduction

Polyploids, organisms with more than two complete sets of chromosomes, are very common among flowering plants and can derive from a mutation in chromosome number (autopolyploids), or from concurrent hybridization and mutation in chromosome number (allopolyploids) (reviewed in [1] and [2]).

Alterations to gene expression and the phenotypic consequences of polyploidy are thought to impact the evolutionary potential of polyploids [3], [1]. Polyploidy has been suggested to affect changes in gene regulation and epigenetic instability [4], [1]. Phenotypic instability and rapid gene silencing, accompanied by DNA methylation re-patterning and transcriptional changes, were found in newly formed *Arabidopsis thaliana* x *Arabidopsis arenosa* allopolyploids [5], [6]. Gene expression changes in *Arabidopsis* allopolyploids were consistent with a RNA-mediated mechanism, which may also play a role in the control of duplicates or homeologous genes in polyploids [7] . Genome-wide activation of suppressed heterochromatic elements is a suggested outcome of regulatory instability (genomic shock, [8]) that characterizes allopolyploids [1], [2]. Evidence consistent [9], [10] and discrepant with this hypothesis [6], [11] has been presented, perhaps owing to the different experimental systems. In contrast to the findings in allopolyploids, limited reports of instability are available for autopolyploids. Transcriptome analysis revealed limited regulatory changes compared to those displayed by hybrids and allopolyploids [7], [12], [13].

Evidence connecting epigenetic changes to genome doubling has been reported in *A. thaliana* [14], [15]. A transgenic locus that was stable in diploids, was frequently silenced in triploid and tetraploid individuals. The silenced allele was stably inherited and, in addition, the allele was paramutagenic, being able to convert an active allele to the silenced state. While this phenomenon indicated that polyploidy enhances epigenetic silencing, it is not known what specific property of this transgene made it responsive to polyploidy. It is possible that silencing pathways themselves are responsive to ploidy level, but there is no data in the literature on this regard.

RNA-mediated gene silencing is a cellular response to dsRNAs that can lead to gene silencing via transcriptional and post-transcriptional mechanisms [16] [17]. Epigenetic changes in polyploids may occur at the transcriptional and post-transcriptional levels [2]. Post-transcriptional gene silencing (PTGS) involves degradation of mRNAs targeted by small interfering RNAs (siRNAs) [18], [19]. PTGS can be induced by production of dsRNA and siRNA from inverted-repeat transgenes [20], [21], [22] and is likely mediated by different dsRNA-processing enzymes (DICER) and RNA-induced silencing complex (RISC), which act redundantly [23], [24], [25]. The interaction of RNA and protein species in the nucleus suggests that nuclear volume or other unknown cellular parameters may affect this interaction.

Little is known about RNA-mediated gene silencing and polyploidy. In the allopolyploid *A. suecica* (hybrid of *A. thaliana* and *A. arenosa*), transgene-induced RNAi has been successfully used as a tool for generating dominant loss-of-function mutations. Expressing the dsRNA corresponding to the *A. thaliana* gene copy was sufficient to silence both the *thaliana* and *arenosa* orthologs. While this study indicates that RNAi can function in polyploids, a direct comparison between matched diploid-tetraploid pairs is necessary to determine efficiency.

In this work, we tested the hypothesis that an increase in ploidy enhances transgene-induced post-transcriptional silencing. We compared the silencing efficiency of endogenous gene mediated by a transgene using experimentally generated *A. thaliana* tetraploids and their progenitor diploids. Our results indicate that a change in ploidy neither enhances nor suppresses the silencing action of dsRNA-generating hairpin transgenes. This provides important information for both the consequences of polyploidy for genome regulation and for the application of PTGS to polyploid species.

## Materials and Methods

### Plant Material

**Figure 1:**
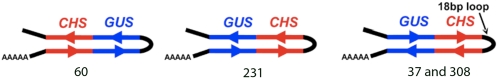
Inverted repeat (hairpin) structure of the expected RNAs produced by CHS transgenic A. thaliana lines. The corresponding gene constructs differ in the position and orientation (indicated by the arrow) of the fragments (CHS and GUS) within the hairpins (modified from Wroblewski et al. 2007-submitted). The transgenic line numbers are indicated below the corresponding RNA.

Transgenic lines of *A. thaliana* ecotype Ws-0 were generated with different inverted repeat constructs driven by the CaMV-35S promoter (Fig. 1) by *Agrobacterium tumefaciens* (strain LBA4404) mediated transformation (floral dip, [26]). All the constructs contained a 342bp fragment of the *chalcone synthase* gene (CHS, GenBank accession number: Y18603, bases 359 to 730) and a 324bp fragment of the GUS gene (GenBank accession number: M14641, bases 2430 to 2771) separated by an intron of the *pyruvate orthophosphate dikinase* gene (*pdk*: GenBank accession number X79095, bases 7890 to 8668). Constructs differed in the position and orientation of the fragments within the hairpins (Fig. 1). After splicing, the *pdk* gene formed the 18bp hairpin loop. Transgenic lines were selected by visually scoring anthocyanin production.

To test transgene insertion locus number and select for lines with a single locus, seeds were surface-sterilized (20% bleach) and planted on an agar-solidified medium containing 1× MS salts, 1% sucrose and 50 mg/ml kanamycin sulfate (pH 6). Seeds were stratified for 4 days in the dark at 4°C and then moved to a growth room under 16 h of artificial daylight (100–150 µE m^−2^ s^−1^) provided with fluorescent bulbs (Philips, F32T8/TL841 UNIVERSAL/HI-VISION, U.S.A.) at 21°C. Four lines, 60, 37, 231 and 308, showing a segregation ratio of 3∶1 (consistent with T-DNA insertion at a single locus; p<0.1), were identified.

The segregation ratio was confirmed by genotyping 20 individuals per segregating line (lines 60, 37, 231 and 308). DNA was extracted from rosette leaves (∼1 g) as previously described [5] and used as template in PCR with primers targeting the *Neomycin phosphotransferase* II gene (NptII, GenBank accession number: AB289803, bases 3025 to 3530) (f 5′-AGGCAGCGCGGCTATCGTGGCTG-3′, r 5′-CTCTTCAGCAATATCACGGGTAG-3′). PCR data were analyzed using chi-square (χ2) test for 1 degree of freedom.

Southern blot was performed to determine the number of transgene copies inserted in each single-locus line. Ten µg of genomic DNA were digested with 100 U of *BamHI*, subjected to electrophoresis on a 0.8% agarose gel and blotted on nylon membrane positively charged (Roche Diagnostic, Indianapolis, U.S.A.). The blot was hybridized at 42°C with a probe corresponding to the NptII gene (same sequence as amplified in the PCR above) and labeled with the DIG High Prime DNA Labeling and Detection Starter Kit I (Roche Applied Diagnostic, Indianapolis, U.S.A.). Hybridization and detection were done following manufacturer's instructions.

Homozygous individuals were generated by selfing kanamycin resistant segregants and confirmed by phenotyping their progeny for kanamycin resistance.

Diploid plants were tetraploidized with a modified protocol from Santos *et al.*, as described previously using 0.25% colchicine [10]. The nuclear DNA content of tetraploid progeny was analyzed by flow-cytometry using propidium iodide fluorescence according to previous reports [27], [28]. Polyploid plants also exhibited a typical increase in flower size characteristic of most polyploids of *Arabidopsis* (Dilkes and Comai, unpublished observations).

### CHS induction and Anthocyanin Assay

For each transgenic line, 18 diploid and 18 tetraploid individuals were planted on soil and incubated at 4°C for 4 days. Diploid and tetraploid Ws-0 wild type plants were used as controls. To score anthocyanin production, plants were grown under 16 h of artificial daylight (see above) at 21°C until the emergence of 5 to 6 true leaves and then grown with 16 h light (900 µE m^−2^ s^−1^) at 21°C, 8 h dark at 14°C to induce CHS gene expression.

Sucrose was also used to induce CHS gene expression and anthocyanin production. Seeds of diploid and tetraploid individuals for each line were surface-sterilized and planted on 6% sucrose plates with minimal medium. Plates were incubated in the dark for 3 days at 4°C and then moved to growth room (16 h artificial daylight at 21°C) for 10 days. Anthocyanin induction was estimated visually and documented by photography.

Anthocyanins were extracted from a rosette leaf with 1% HCl methanol as described by Davies *et al*. [29]. Nine plants were assayed per line and ploidy level. Anthocyanin content was quantified using a plate reader (SpectraMax 190- Molecular Devices). OD_530_ readings were normalized to leaf fresh weight. The normalized OD_530_ values were compared between diploids and tetraploids within each transgenic line by Student's t-test [30]. The role of ploidy within the entire population of transgenic lines was estimated by regression using the formula OD_530_ = line+ploidy+e where line and ploidy are categorical variables and e corresponds to the error.

### RNA isolation and RT real-time PCR

Total RNA was extracted from seedlings (∼100 mg, two biological replicates) grown on 1% sucrose plates with the RNeasy Mini Kit (Qiagen Inc., Valencia, CA). Three µg of RNA were DNAse-treated according to the manufacturer's instructions in a 20 µl reaction (Invitrogen, Carlsbad, CA). For cDNA synthesis, 5 µl of DNAse-treated total RNA were incubated with 1.5 µl of random hexamers (Invitrogen, Carlsbad, CA) for 5 minutes at 70°C. Reverse-transcription (RT) mixture was added, with a final concentration of 1× Buffer, 20 µM dNTPs, 4mM DTT, 20U of MMLV reverse transcriptase (Invitrogen, Carlsbad, CA) and was incubated for 1 hour at 42°C.

SYBR Green real-time PCR was used to quantify CHS mRNA expression by the comparative threshold cycle method (User Bulletin #2; Applied Biosystem, Foster City, CA). Primers for CHS mRNA quantification (f 5′-TGCTCTGAGATCACAGCCGTTA -3′, r 5′-CCGACGAGGGAGTCAAGGT-3′) were designed outside the silencing region, using the Primer Express software (Applied Biosystems). 18s rRNA was adopted as the endogenous control (f 5′-TGCAACAAACCCCGACTTATG-3′, r 5′-CCCGCGTCGACCTTTTATC-3′), as previously proposed [31]. Real-time PCR reactions (25 µl) were done in 96 well reaction plates adding 1× SYBR Green PCR Master Mix (Applied Biosystems), 5 µl cDNA and 400 nM primers. Three technical replicates were tested for each sample. Real-time PCR was performed in an ABI Prism 7500 Sequence Detection System under standard amplification conditions. Melting curve analysis was performed for each gene (CHS, 18s rRNA).

## Results

### Identification of single locus CHS inverted repeat transgenic lines

Transgenic *Arabidopsis thaliana* (ecotype Ws-0) lines were generated by transformation with inverted repeat sequences of *chalcone synthase* (CHS) and GUS sequences to produce chimeric interfering hairpin RNAs (Fig. 1) for transgene-induced silencing of the endogenous CHS gene. Based on their anthocyanin accumulation phenotypes, we selected 20 transgenic lines for further characterization.

Segregation of the Kanamycin resistance phenotype was used to identify lines segregating for a single T-DNA insert location. The selfed progeny from four lines (designated 60, 37, 231, 308) out of the 20 segregated consistent with the 3∶1 ratio, as expected for insertion at a single locus in the plant genome. Genotyping 20 individuals per segregating line further confirmed the 3∶1 segregation ratio (χ^2^ values = 0.5–0.8; p>0.1). Insert copy number at each single locus was determined by Southern blot (data not shown). Lines 60 and 37 displayed multiple hybridization-positive bands (4 and 2 respectively), while lines 231 and 308 displayed a single positive band. The matched diploid-tetraploid lines thus differed only by the number of chromosomes, and thus by the number of transgenic alleles (2 versus 4). Homozygous individuals were obtained by selfing. We generated tetraploid individuals by colchicine treatment of the diploid homozygous individuals. We confirmed ploidy in the selfed progeny of colchicine-treated plants by phenotype and flow cytometry (data not shown).

### Ploidy level does not influence the efficiency of transgene induced CHS gene silencing

**Figure 2:**
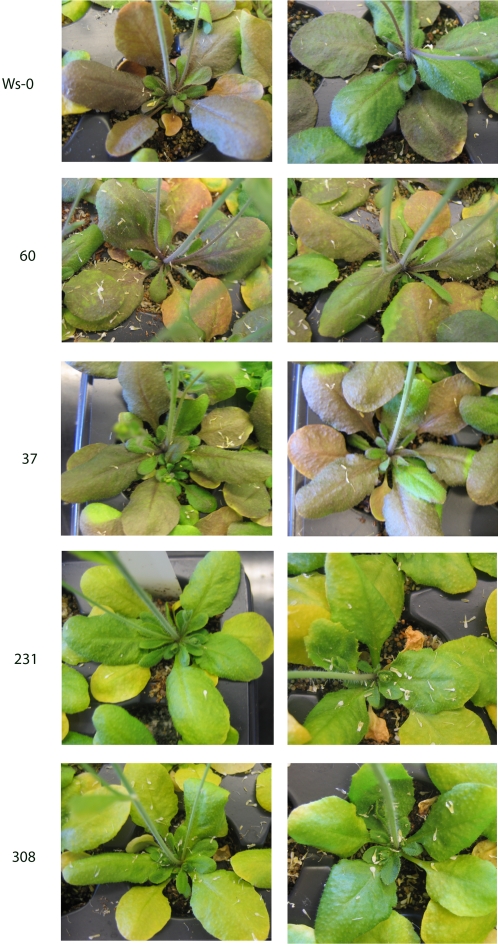
Anthocyanin accumulation in rosette leaves of wild-type A. thaliana Ws-0 and CHS hairpin interfering transgenic lines. Diploids (left) and tetraploids (right) were incubated in CHS inducing conditions for 15 days. The transgenic line numbers are indicated on the left.

In order to determine the effect of ploidy on gene silencing, we compared the efficiency of CHS gene silencing in diploid and tetraploid individuals of transgenic lines 60, 37, 231, 308 grown under the same CHS-inducing conditions. UV-B and UV-A irradiance and low temperature were used to induce CHS expression as described in previous literature [32]. After 10 days of incubation, the adaxial surface of rosette leaves from wild-type Ws-0 and lines 60 and 37 started to turn purple in response to CHS expression (Fig. 2). We did not observe a difference between diploid and tetraploid individuals. In the case of lines 231 and 308 lines leaf color of both diploids and tetraploids remained green (Fig. 2) indicating strong silencing of CHS by the transgene in these lines. Thus, two lines out of four (line 60 and line 37) showed a weak silencing phenotype and two lines (line 231 and line 308) appeared to be very effective at silencing CHS in both diploid and tetraploid individuals. The use of these four lines allowed us to determine the effect of ploidy on hairpin inverted repeat silencing across a range of silencing efficiencies. No difference was discerned between diploid and tetraploid lines by visual inspection. This suggests that ploidy level does not influence the efficiency of inverted-repeat induced gene silencing.

**Figure 3:**
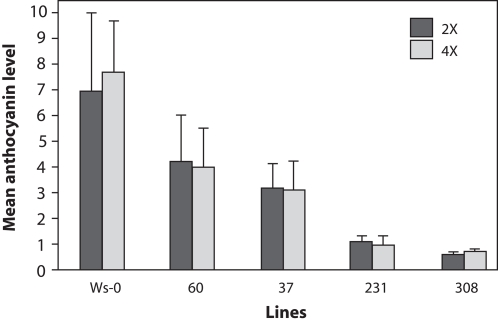
Anthocyanin quantification in transgenic plants, compared to wild-type Ws-0. Anthocyanin levels (Y axis) are expressed as the mean of OD530 readings for 9 biological replicates. Genotype is indicated on the X-axis. Error bars indicate the standard deviation. In the legend: 2x is diploid, 4x tetraploid.

To confirm these observations, one leaf from nine plants from each ploidy level and line was sampled and anthocyanin extraction was performed using acidified methanol. Anthocyanin levels, as measured by OD_530_ readings, are shown in Fig. 3. As expected, lines 60 and 37 showed higher accumulation of anthocyanin than the other transgenic lines, but were remarkably lower when compared to wild-type plants. For lines 231 and 308, very little anthocyanin was detected. Comparisons of OD_530_ values between diploid and tetraploid individuals belonging to the same line by Student's t-test were not significant at p<0.05 (Fig. 3). To test for a more subtle effect of ploidy on transgene-induced silencing, regression analysis was used to test for the effect of ploidy within the entire population assayed. Ploidy was not significantly associated with variation in OD_530_ levels in the entire population (data not shown).

### Ploidy level does not affect CHS mRNA accumulation

**Figure 4:**
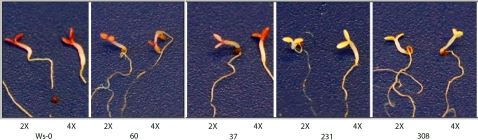
Photograph of seedlings showing anthocyanin accumulation. Seedlings were grown on 6% sucrose medium for 15 days. The transgenic line numbers are indicated below the photograph. 2x is diploid, 4x tetraploid.

To confirm the results obtained by light and temperature induction of anthocyanins, seedlings of diploids and tetraploids were grown on 6% sucrose plates to induce CHS mRNA accumulation [33]. In addition to CHS accumulation, chlorophyll biosynthesis is inhibited by these conditions, resulting in the absence of green tissue. This experiment allows discrimination between wild-type seedlings, which appear purple because of anthocyanin accumulation induced by the high concentration of sucrose in the medium, and white seedlings where CHS has been silenced. After 10 days of incubation in a growth chamber, the phenotype of seedlings was scored. Wild-type Ws-0 seedlings were purple, while those of lines 60 and 37 were faint purple. Lines 231 and 308 seedlings appeared white (Fig. 4).

**Figure 5:**
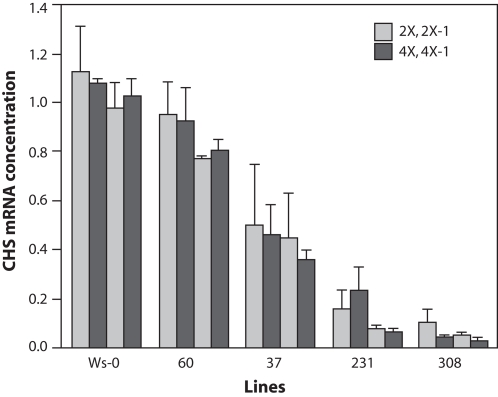
Quantification of CHS mRNA in transgenic seedlings. Seedlings were grown for 15 days on 6% sucrose medium and then sampled. SYBR Green Real-time RT-PCR was performed with the comparative threshold method, using 18s rRNA as endogenous control. The Y-axis displays the mean of three technical replicates in each of two biological replicates. Genotype is indicated in X-axis. In the legend: 2x and 2x-1 is diploid, 4x and 4x-1 tetraploid.

Seedlings were harvested and used to quantify CHS mRNA by real-time RT PCR analysis. Wild-type Ws-0 plants showed the greatest CHS mRNA accumulation and were used to normalize values from the other lines. Diploids of lines 60 and 37 accumulated substantial CHS mRNA, with line 60 accumulating between 78% and 95% of the wild-type CHS mRNA concentration. Diploids from lines 231 and 308 showed extremely low CHS mRNA accumulation (Fig. 5) accumulating less than 15% and 9% respectively. Results of sucrose induction and real-time PCR were in agreement and confirmed the weak (lines 60 and 37) and strong (lines 231 and 308) CHS silencing efficiencies observed previously with the anthocyanin assay. Moreover, no difference in the level of CHS mRNA induction was detected between diploid and tetraploid individuals from the same line indicating no effect of ploidy level on CHS mRNA accumulation in seedlings.

## Discussion

We tested the hypothesis that an increase in ploidy could enhance transgene-induced post-transcriptional gene silencing by comparing silencing efficiency on the endogenous *chalcone synthase* gene (CHS) between diploids and tetraploids of four independent *A. thaliana* lines transformed with interfering hairpin transgenes. We used homozygous transgenic lines: as a result, the polyploids have four transgenic alleles instead of the two present in homozygous diploids. The ratio of transgene alleles to target locus alleles, however, remains unchanged at 1∶1. We reasoned that if absolute copy number, or mutually reinforcing allelic interactions influence silencing efficiency, tetraploids would more readily down-regulate the endogenous CHS target. Alternatively, an increase in ploidy might have hindered the action of the transgenes perhaps by altering special relationships in the nucleus. Both an increase or a decrease in silencing efficiency determined by increased ploidy would be manifest as a change in the amount of anthocyanin accumulation and a concomitant change in mRNA levels of the target CHS. Increases would have been most obvious in the partially silenced lines 60 and 37. Decreases would have been most obvious in the strongly silenced lines 231 and 308. Yet, we found no difference in silencing efficiency between diploids and tetraploids. We conclude that there is no support for an effect of ploidy level on epigenetic regulation via PTGS.

Our results differ from, but are not inconsistent with the observation of ploidy-dependent paramutation in *A. thaliana* [14], [15], where ploidy increased transcriptional gene silencing. The different observations may be explained by considering the action of different silencing mechanisms in the transgene-induced paramutation and in our hairpin-induced silencing of CHS. Collectively, the existing evidence and the results presented here suggest that future inquiries on ploidy-dependent regulatory effects should focus on mechanisms that regulate transcriptional responses.

Another implication of our work is that transgenic PTGS approaches aimed at basic and applied studies should be expected to have comparable effects regardless of ploidy level. This is important as silencing work in plants has involved the use of diploid and tetraploid species and the question of whether PTGS is comparable in these two systems was unanswered until now. In addition, many important crop species such as potato are autotetraploids and many more are allopolyploids. The demonstration that transgene-induced silencing is as efficient in tetraploids as in diploids of *Arabidopsis* will thus facilitate the interpretation of experiments in which polyploids are used to study gene function or to modify crop properties.
